# Artificial Intelligence-Derived Electrocardiogram Analysis for Identification of Carbon Monoxide-Induced Cardiomyopathy: A Retrospective Study

**DOI:** 10.3390/medicina62061081

**Published:** 2026-06-02

**Authors:** Heewon Yang, Moon-Seung Soh, Min Sung Lee, Sungwoo Choi, Sangsoo Han, Sung-Eun Lee, Yura Ko, Sangchun Choi

**Affiliations:** 1 Department of Emergency Medicine, Ajou University School of Medicine, Suwon 16499, Republic of Korea; speedheewon@aumc.ac.kr (H.Y.); plumpboy@aumc.ac.kr (S.-E.L.); 109427@aumc.ac.kr (Y.K.); 2 Department of Cardiology, Ajou University School of Medicine, Suwon 16499, Republic of Korea; 108968@aumc.ac.kr; 3 Medical AI Co., Ltd., Seoul 06180, Republic of Korea; lylm@medicalai.com; 4 Digital Healthcare Institute, Sejong Hospital, Bucheon 14754, Republic of Korea; 5 Department of Emergency Medicine, Soonchunhyang University College of Medicine, Seoul 04401, Republic of Korea; 121791@schmc.ac.kr (S.C.); brayden0819@schmc.ac.kr (S.H.); 6 Department of Neurology, Ajou University School of Medicine, Suwon 16499, Republic of Korea

**Keywords:** artificial intelligence, electrocardiogram, biomarker, carbon monoxide poisoning, cardiomyopathy

## Abstract

*Background and Objectives*: The diagnostic accuracy of an artificial intelligence (AI)-derived initial 12-lead electrocardiogram (ECG) analysis was evaluated for early carbon monoxide-induced cardiomyopathy (CO-CMP) risk detection. *Materials and Methods*: Retrospective medical data of carbon monoxide poisoning (COP) cases between 1 January 2015 and 31 December 2024 were screened for the primary outcome: odds ratio (OR) for echocardiographically confirmed CO-CMP among those with high-risk probability score per the AI-derived model. Secondary outcomes included left ventricular ejection fraction (LVEF) and AI-derived probability score, critical care requirements, including intubation and intensive care unit (ICU) admission, and cardiac arrest events. *Results*: A total of 51 patients with acute COP were included in the final analysis, with 13 (25.5%) being diagnosed with CO-CMP. The LVEF in the CO-CMP group was lower than that in the non-CO-CMP group (40.00 ± 13.80% vs. 63.76 ± 6.24%, *p* < 0.001). The AI-derived probability score was higher in the CO-CMP group (11.3 [3.8–32.7] vs. 0.5 [0.2–2.2], *p* < 0.001). Among cardiac biomarkers, troponin I (2.37 [0.32–7.88] vs. 0.06 [0.06–0.95] ng/mL, *p* = 0.002) was higher in the CO-CMP group. Patients with CO-CMP required recurrent ventilator support (76.9% vs. 21.1%, *p* < 0.001) and ICU admission (92.3% vs. 42.1%, *p* = 0.003). In multivariable regression analysis, the AI-derived prediction model was independently associated with CO-CMP (OR 1.14; 95% confidence interval (CI) 1.02–1.27; *p* = 0.017; Firth-penalized OR 1.11; 95% CI 1.03–1.25; *p* < 0.001). Receiver operating characteristic analysis of the AI-derived model showed an area under the curve of 0.85 (95% CI 0.70–0.96) for the AI score alone and 0.92 (95% CI 0.83–0.99) for the Combined AI–cardiac marker model, with a sensitivity of 92.3% and specificity of 81.6%. Pairwise DeLong comparisons between the Combined AI model and comparator models did not reach statistical significance (Combined vs. AI-only, *p* = 0.092; Combined vs. cardiac markers, *p* = 0.052); however, the likelihood-ratio test for adding the AI probability score to the cardiac marker-only model demonstrated significant incremental information (χ^2^ = 13.68, *p* < 0.001). *Conclusions*: AI-based ECG analysis showed exploratory diagnostic association with LV systolic dysfunction observed in suspected CO-CMP patients. Given the limited sample size, low events-per-variable ratio, and lack of external validation, these findings suggest that AI-ECG analysis may provide incremental information for early cardiac risk stratification in selected patients.

## 1. Introduction

Carbon monoxide (CO) poisoning (COP) is a life-threatening emergency condition characterized by systemic hypoxia and cellular toxicity following exposure to CO [1]. CO is a colorless, odorless gas with an affinity for hemoglobin 200–250 times greater than that of oxygen, thereby impairing oxygen delivery and inhibiting mitochondrial cytochrome c oxidase, which disrupts oxidative phosphorylation and induces cellular injury [2,3]. COP represents a substantial global health burden and remains a leading cause of poisoning-related mortality worldwide, with a particularly significant impact reported across Asian countries [4,5].

The clinical manifestations of COP correlate with blood carboxyhemoglobin (CO-Hb) levels. At concentrations of 30–50%, patients typically present with dizziness, confusion, vomiting, and syncope, often accompanied by tachycardia and tachypnea; seizures may occur at levels of approximately 40%. When CO-Hb exceeds 50%, the risks of seizures, coma, severe metabolic acidosis, and death increase substantially, with levels ≥ 60% being frequently fatal [6]. These systemic effects result in significant neurological and cardiovascular toxicity. Among end organs, the heart is particularly vulnerable, with arrhythmias, myocardial ischemia, and left ventricular dysfunction reported even at CO-Hb levels of approximately 20%, a range commonly encountered in the emergency department (ED). In this context, CO-induced cardiomyopathy (CO-CMP) represents one of the most severe and clinically consequential complications of COP and is a major contributor to morbidity and mortality [7]. However, the early identification of CO-CMP in the ED remains challenging. Delayed recognition of CO-CMP may result in missed opportunities for timely hemodynamic support and appropriate cardiac monitoring, potentially leading to worse clinical outcomes. Electrocardiographic abnormalities in COP are frequently either nonspecific or transient, whereas elevations in conventional cardiac biomarkers, such as troponin I or troponin T and creatine kinase myocardial band (CK-MB), may be delayed or lack sufficient diagnostic specificity, thereby limiting their utility during the initial ED evaluation [8,9,10,11]. Although echocardiographic parameters, including left ventricular global longitudinal strain, have been shown to predict CO-CMP, echocardiography is often not immediately available or feasible during early emergency care [8].

Recent advances in artificial intelligence (AI)-enabled electrocardiogram (ECG) analysis may support early risk stratification of cardiomyopathies and left ventricular systolic dysfunction (LVSD) [12,13,14]. AI-derived ECG models have also shown an ability to detect subclinical cardiac abnormalities, such as reduced ejection fraction and silent atrial fibrillation, using standard 12-lead ECGs obtained at presentation, thereby highlighting their potential role as rapid, non-invasive decision-support tools in the ED [15]. As CO-CMP often manifests with subtle or transient electrocardiographic changes, AI-enabled ECG analysis may be particularly well-suited to detect latent myocardial dysfunction that is not readily apparent on conventional ECG interpretation.

Given the diagnostic challenges of CO-CMP and the time-sensitive nature of myocardial injury in COP, we hypothesized that an AI-derived analysis of the initial 12-lead ECG obtained in the ED could enable accurate CO-CMP detection. Accordingly, this study aimed to evaluate the diagnostic performance of an AI-based 12-lead ECG as an early ED-based digital adjunct for CO-CMP, compared with conventional biomarkers, including troponin I, and CO-Hb levels. Using the Medical AI’s pre-specified threshold of AI score ≥ 9.7, AiTiALVSD identified 9 of 51 patients (17.6%) as positive, sensitivity 53.8% (95% CI 29.1–76.8), specificity 94.7% (82.7–98.5), PPV 77.8%, NPV 85.7%, and accuracy 84.3% for the broader CO-CMP outcome. At the data-driven Combined model operating point (predicted probability ≥ 0.15), sensitivity rose to 92.3% (95% CI, 63.2–96.9) at specificity 81.6% (95% CI, 66.6–90.8). Two robustness analyses were performed to assess the stability and potential overfitting of the exploratory Combined AI model. First, Firth-penalized logistic regression was applied because of the limited number of outcome events and the low events-per-variable ratio. In this analysis, the AI-derived probability score remained the only independent predictor of CO-CMP (penalized OR 1.11; 95% CI, 1.03–1.25; penalized likelihood ratio *p* < 0.001), whereas conventional cardiac biomarkers were not independently associated with the outcome. Second, bootstrap internal validation with 1000 resamples was performed for the exploratory Combined model. The apparent AUC was 0.921, with a mean optimism estimate of 0.052, corresponding to an optimism-corrected AUC of 0.87 (95% bootstrap percentile CI, 0.71–1.00). These findings suggest that the apparent diagnostic performance was partly influenced by optimism and potential overfitting, particularly given the limited sample size and low events-per-variable ratio. Therefore, the Combined model should be interpreted as exploratory and hypothesis-generating rather than as a definitive clinical prediction model, and external validation in a larger multicenter cohort is warranted. Detailed results are provided in Supplementary Table S2.

## 2. Materials and Methods

### 2.1. Study Design and Data Collection

This retrospective observational study was conducted at a single tertiary emergency medical center in South Korea. The study protocol was approved by the Institutional Review Board of the authors’ university hospital (approval number AJOUIRB-DB-2025-256) and was conducted in accordance with the Declaration of Helsinki. The requirement for informed consent was waived due to the retrospective nature of this study.

We collected the medical records of patients who were diagnosed with CO poisoning and presented to the ED between 1 January 2015 and 31 December 2024, and underwent electrocardiography and cardiac biomarker testing (troponin I and CK-MB) within 1 h of ED arrival.

For all patients, the first 12-lead ECG obtained <1 h after ED presentation was analyzed. Patients were eligible for inclusion if they had confirmed COP and suspected cardiac involvement. Elevated troponin I was defined as a level exceeding the 99th percentile upper reference limit (≥0.04 ng/mL) based on the institutional assay. Suspected cardiac involvement was defined as patients who underwent echocardiography because of new electrocardiographic abnormalities (ST-segment changes, T-wave inversion) or persistent cardiac symptoms such as chest pain or dyspnea following CO exposure.

The primary outcome was the odds ratio (OR) for echocardiographically confirmed CO-CMP among patients classified as high-risk according to the AI-based ECG prediction model. Secondary outcomes included echocardiographic left ventricular ejection fraction (LVEF, %), clinical characteristics (intentional poisoning, hypertension, obstructive coronary artery disease, congestive heart failure, and suicidal intent), critical care requirements (endotracheal intubation and intensive care unit (ICU) admission), and the occurrence of cardiac arrest. Laboratory variables analyzed included troponin I, CO-Hb concentration, blood urea nitrogen (BUN), and serum creatinine (Cr).

Patients were not classified into the suspected cardiac injury group if all of the following applied at initial ED evaluation: (1) no persistent cardiopulmonary symptoms; (2) normal initial troponin I; (3) no ECG changes; and (4) no cardiologist consultation for official echocardiography during admission. Conversely, patients with any of these risk features who underwent echocardiography within 24 h of ED arrival were considered to have suspected cardiac involvement and were eligible for AiTiALVSD analysis.

### 2.2. Definition of CO-CMP

CO-CMP was defined on transthoracic echocardiography as the presence of at least one of the following abnormalities: reduced left ventricular systolic function, defined as an LVEF < 50%; newly developed regional wall-motion abnormality; or impaired right ventricular systolic function, as determined by standard echocardiographic criteria [16,17,18,19]. According to the institutional protocol of our poisoning center, transthoracic echocardiography was routinely performed within 24 h of CO exposure in patients with suspected cardiac involvement. Echocardiographic findings were interpreted by board-certified cardiologists who were blinded to the AI-derived ECG results. All patients diagnosed with CO-CMP were further evaluated through cardiologist consultation to exclude pre-existing or existing structural heart disease or cardiovascular conditions that could independently cause myocardial dysfunction.

### 2.3. AI-Based ECG Model

In this study, the patients’ initial 12-lead ECGs were obtained upon presentation to the ED, within 1 h of arrival. AiTiALVSD (Medical AI Co., Ltd., Seoul, Republic of Korea) is a deep-learning-enabled Software as a Medical Device approved by the Ministry of Food and Drug Safety of the Republic of Korea in 2023 for screening left ventricular systolic dysfunction (LVSD) from 12-lead ECGs. The algorithm accepts digital 12-lead raw ECG signals sampled at 500 Hz as input, without incorporating additional clinical variables, and generates an LVSD probability score ranging from 0 to 100. A pre-established threshold of 9.7 is used to classify patients as high or low risk, calibrated to achieve approximately 90% sensitivity [20]. Based on prior validation studies, a pre-specified cutoff value of 9.7 was applied to dichotomize the results into high- and low-probability categories. The AI-ECG predictions were then compared with actual echocardiographically measured LVEF for diagnostic performance assessment. We evaluated these clinical outcomes as three distinct methods: (1) AiTiALVSD threshold (probability score ≥ 9.7), pre-specified and externally validated by the manufacturer for screening of left ventricular systolic dysfunction (LVEF). (2) AI analysis threshold derived in the present cohort by maximizing Youden’s J against the study outcome. (3) Combined model operating point (predicted LVEF cutoff), output of multivariable logistic regression integrating AI score with cardiac biomarkers (troponin I and CK-MB), with the cutoff again selected by Youden’s J on the present cohort.

### 2.4. Statistical Analysis

Continuous variables are expressed as the mean ± standard deviation or median with interquartile range. Categorical variables are presented as percentages. Normality was assessed using the Shapiro–Wilk test and visual inspection of Q–Q plots. Comparisons between two groups were performed using Student’s *t*-test for continuous variables and the Chi-squared test for categorical variables. Non-normally-distributed variables are expressed as the median [interquartile range (IQR)] and were compared using the Mann–Whitney U test. The AI-derived probability score was included as the primary predictor of interest. Troponin I, CO-Hb, and CK-MB were included as established biomarkers associated with myocardial injury. For multivariable analysis of a binary outcome, logistic regression was used to estimate ORs with 95% confidence intervals (CIs). The discriminative ability of continuous predictor variables was evaluated using receiver operating characteristic (ROC) curve analysis, and the area under the ROC curve (AUC) was reported. The analysis for troponin I and CK-MB was performed using negated values (troponin I, CK-MB) to account for the directionality of each biomarker. The optimal cut-off value was selected based on the maximal Youden index, defined as Youden’s J = sensitivity + specificity − 1. All statistical tests were two-tailed, and a *p*-value < 0.05 was considered statistically significant. In order to address the limitation of the small sample size, two pre-specified robustness analyses were performed: Firth’s penalized logistic regression and non-parametric bootstrap internal validation (1000 resamples) [21,22]. Additionally, a pre-specified sensitivity analysis restricted to patients with isolated left ventricular systolic dysfunction (LVEF < 50%) was performed to evaluate the robustness of the findings relative to the intended use of the AiTiALVSD algorithm. The manufacturer-defined threshold analysis (AI score ≥ 9.7) was considered the primary hypothesis-driven analysis because this cutoff had been externally validated for LVSD screening. Cohort-derived cutoffs and Combined model operating thresholds were considered exploratory secondary analyses. All statistical analyses were performed using R software, version 4.6.0 (R Foundation for Statistical Computing, Vienna, Austria).

## 3. Results

**Figure 1:**
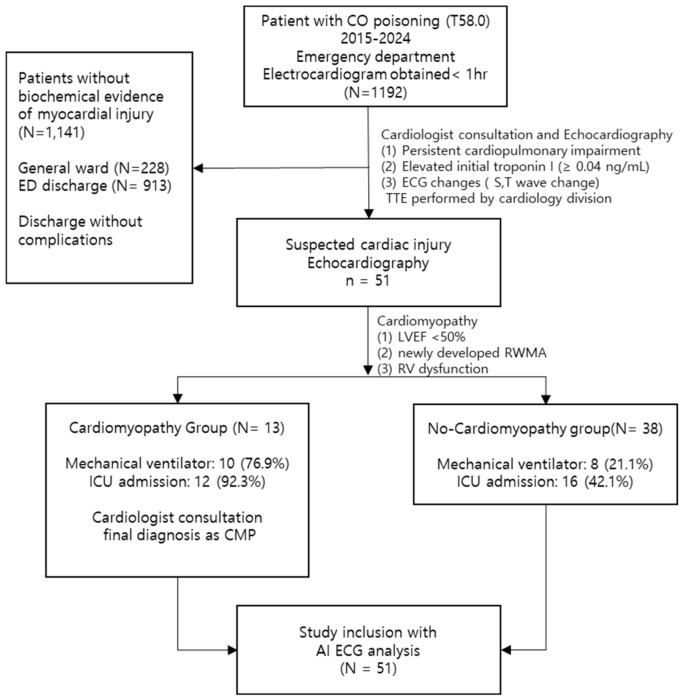
Flow diagram of patient inclusion and study design.

Between January 2015 and December 2024, a total of 1192 patients diagnosed with carbon monoxide poisoning (ICD-10: T58.0) who had an initial 12-lead ECG obtained within 1 h of emergency department (ED) presentation were identified. Of these, 1141 patients did not meet criteria for suspected cardiac injury and were excluded from further analysis; 913 were discharged directly from the ED, and 228 were admitted to the general ward without suspected cardiac involvement and discharged without any complications. The remaining 51 patients underwent transthoracic echocardiography by a cardiologist for suspected myocardial dysfunction and constituted the final study cohort. A total of 51 patients with acute COP were included in the final analysis, of whom 13 (25.5%) were diagnosed with CO-CMP (Table 1 and Figure 1). There were no significant differences between the CO-CMP and non-CO-CMP groups in age, sex, or prevalence of hypertension. Intentional CO exposure was significantly more frequent in the CO-CMP group (92.3% vs. 47.4%, *p* = 0.005).

LVEF was lower in the CO-CMP group than in the non-CO-CMP group (40.0 ± 13.8% vs. 63.8 ± 6.2%, *p* < 0.001). The AI-derived probability score was higher in the CO-CMP group (11.3 [3.8–32.7] vs. 0.5 [0.2–2.2], *p* < 0.001). The levels of troponin I (2.37 [0.32–7.88] vs. 0.06 [0.06–0.95] ng/mL, *p* = 0.002) and CK-MB (26.6 [16.6–149.0] vs. 2.2 [0.5–21.0] ng/mL, *p* < 0.001) were higher in the CO-CMP group. Additionally, BUN (21.0 [16.8–35.0] vs. 15.0 [12.6–21.0] mg/dL, *p* = 0.011) and creatinine levels (1.7 [1.2–2.4] vs. 0.9 [0.7–1.1] mg/dL, *p* < 0.001) were also elevated in the CO-CMP group. Patients in the CO-CMP group required more mechanical ventilatory support (76.9% vs. 21.1%, *p* < 0.001) and ICU admission (92.3% vs. 42.1%, *p* = 0.003).

**Figure 2:**
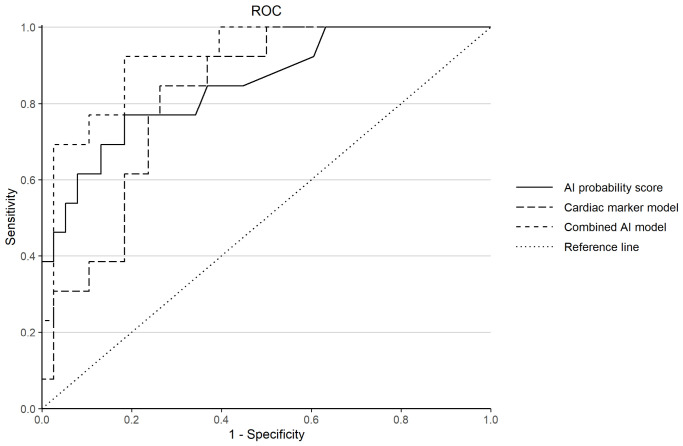
Receiver operating characteristic curves for the prediction of carbon monoxide-induced cardiomyopathy. The solid line shows the AI probability score alone (AUC 0.85; 95% CI, 0.70–0.96). The long-dashed line represents the Cardiac marker model (troponin I + CK-MB; AUC 0.82; 95% CI, 0.70–0.94). The short-dashed line represents the Combined AI model (AI probability score + troponin I + CK-MB; AUC 0.92; 95% CI, 0.83–0.99). The Combined AI model demonstrated higher diagnostic accuracy compared with the cardiac marker model alone (DeLong test, *p* = 0.052) and the AI probability score alone (*p* = 0.092). ROC, receiver operating characteristic; AUC, area under the curve; CI, confidence interval; AI, artificial intelligence; CK-MB, creatine kinase myocardial band.

In the multivariable binary logistic regression analysis with the combined AI model, the AI-derived probability score was independently associated with CO-CMP (OR 1.14; 95% CI, 1.02–1.27; *p* = 0.017), whereas troponin I and CK-MB were not significantly associated with CO-CMP. ROC curve analysis showed that the Combined AI model incorporating the AI probability score, troponin I, and CK-MB achieved the highest diagnostic accuracy (AUC 0.92, 95% CI, 0.83–0.99) compared with the AI probability score alone (AUC 0.85, 95% CI, 0.70–0.96) and the Cardiac marker-only model combining troponin I and CK-MB (AUC 0.82, 95% CI, 0.70–0.94). Pairwise DeLong comparisons between the Combined model and comparator models did not achieve statistical significance (Combined vs. AI-only, *p* = 0.092; Combined vs. Cardiac marker-only, *p* = 0.052). The likelihood ratio test for adding the AI probability score to the Cardiac marker-only model demonstrated significant incremental information (χ^2^ = 13.68, *p* < 0.001), supporting the added value of AI-ECG information beyond conventional cardiac markers (Table 2). Using a Youden-optimal cutoff of predicted probability ≥ 0.15, the Combined AI model yielded a sensitivity of 92.3%, specificity of 81.6%, and Youden index of 0.74 (Figure 2). The positive and negative predictive values of the Combined AI model were 63.2% and 96.9%, compared with 58.8% and 91.2% for the AI probability score alone, respectively.

## 4. Discussion

CO binds to hemoglobin with approximately 200-fold greater affinity than does oxygen, forming CO-Hb and impairing tissue oxygen delivery [23]. As myocardial tissue has a high metabolic demand, CO-induced hypoxia, together with direct mitochondrial inhibition, predisposes cardiomyocytes to injury [24]. Although CO-Hb is the primary indicator of COP, its concentration falls rapidly after the cessation of exposure or administration of oxygen, which implies that delayed measurement may produce a false-negative result [25,26]. Furthermore, conventional indicators of cardiotoxicity, such as troponin elevation or electrocardiographic abnormalities, often lack specificity for CO-related myocardial injury, complicating early diagnosis in the emergency setting.

Recent studies have demonstrated that AI-enabled ECG analysis can detect subtle electrophysiological signatures associated with a wide spectrum of cardiomyopathies, including hypertrophic, dilated, and stress-induced cardiomyopathy, beyond isolated LVSD [13,14,15,27,28]. These AI-derived ECG models extract clinically meaningful information not readily apparent on conventional ECG interpretation and may precede overt structural or functional abnormalities detectable by echocardiography, supporting their potential role as an early digital adjunct for detecting myocardial involvement.

Stress-induced CMP shares important pathophysiological mechanisms with CO-CMP, including acute catecholamine surges, myocardial stunning, and transient myocardial injury [18,29,30]. In this context, our findings suggest that AI-derived ECG probability scores may be particularly well-suited for the early identification of CO-CMP. By capturing stress-related myocardial electrophysiological changes, AI-ECG analysis may enable rapid bedside detection and early risk stratification in acute clinical settings, such as the ED, potentially mitigating downstream morbidity and mortality associated with delayed recognition of CO-related cardiac injury.

The electrophysiologic alterations observed in CO poisoning are biologically plausible given the multifactorial mechanisms of CO-mediated myocardial injury. In addition to impairing oxygen delivery through high-affinity binding to hemoglobin and myoglobin, CO directly inhibits mitochondrial cytochrome oxidase activity, resulting in impaired oxidative phosphorylation, ATP depletion, and increased oxidative stress [20,31]. These metabolic and inflammatory disturbances may contribute to altered myocardial depolarization and repolarization, thereby generating subtle ECG abnormalities that may not be readily recognizable on conventional visual interpretation [32]. Prior studies have also suggested that CO exposure may promote arrhythmogenic electrical instability through catecholamine-mediated stress responses, altered calcium handling, and modulation of ion-channel activity, including late sodium current augmentation and impaired repolarization reserve [20,31,32]. Although our study was not designed to directly investigate these mechanisms, such pathophysiological processes may explain why AI-enabled ECG analysis can detect clinically meaningful electrophysiologic signatures associated with CO-induced myocardial dysfunction.

According to the previous study of Satran D et al., the COP patients with suspected myocardial injury had cardiac troponin I levels over 0.7 ng/mL [32]. In our cohort, the median troponin I level was markedly higher in patients with CO-CMP than in those without CO-CMP. Troponin I was 2.37 [0.32–7.88] ng/mL and 0.06 [0.06–0.95] ng/mL, respectively. In contrast, patients who were clinically stable enough to be discharged from the emergency department or admitted to a general ward generally had troponin I levels within the normal range (<0.04 ng/mL). Therefore, although the lack of echocardiographic assessment in all CO poisoning patients remains an important limitation, the study population reflects the clinically relevant subgroup in whom CO-CMP is actually suspected and evaluated in practice.

In our cohort, CO-CMP was identified in 25.5% of patients, all of whom had intentional CO exposure. Patients with CO-CMP required mechanical ventilation and ICU admission significantly more frequently than those without cardiomyopathy. This highlights the strong association between CO-induced myocardial dysfunction, critical illness severity, and increased utilization of intensive healthcare resources.

Furthermore, the CO-CMP group demonstrated significantly higher AI-ECG probability scores and elevated cardiac biomarker levels. Importantly, among patients with elevated troponin levels prior to echocardiography, the AI-ECG probability score independently predicted CO-CMP; in contrast, troponin I, and CK-MB were not independently associated with the condition. The odds of developing CO-CMP were more than 20-fold higher in the AI-predicted high-risk group compared to the low-risk group. Additionally, ROC analysis revealed that the AI score exhibited superior discriminative ability for CO-CMP compared to conventional biomarkers.

The AiTiALVSD algorithm was originally trained and regulatory-validated for the detection of LVSD, which represents a different spectrum from CO-induced myocardial injury. In a prior external validation cohort, the manufacturer’s pre-specified threshold of 9.7 demonstrated a sensitivity of approximately 80% and an AUC of 0.872 for LVSD detection. Sensitivity analysis restricted to patients with isolated left ventricular systolic dysfunction (LVEF < 50%) demonstrated generally consistent directional results with the primary composite CO-CMP analysis (Supplementary Table S3). The AI-derived probability score remained associated with LV systolic dysfunction, although the effect estimates were attenuated because of the reduced sample size of the restricted subgroup (OR 1.06, 95% CI 1.02–1.13, *p* < 0.001).

False-negative cases of the AI-ECG algorithm were also observed within our cohort. Two such cases are detailed below (Supplementary Table S1, Patients 1 and 9). In the first case (Patient 1), a middle-aged woman, was found unresponsive after intentional charcoal-briquette inhalation in an enclosed indoor space and presented within approximately one hour of exposure (admission CO-Hb 26.3%; troponin I 0.317 ng/mL; CK-MB 25.2 µg/L; CK 1673 U/L). The admission electrocardiogram demonstrated normal sinus rhythm without ST-segment changes. Despite biomarker evidence of myocardial injury, the AI-ECG probability score was only 0.4 (well below the manufacturer-specified threshold of ≥9.7), and the algorithm misclassified the patient as low-risk; the Combined AI model probability (0.10) likewise fell below its operating cutoff. Echocardiography demonstrated focal apical-septal akinesia with preserved global function (LVEF 55%), fulfilling our pre-specified composite definition of CO-CMP. Regional wall-motion abnormalities (RWMA) resolved completely after hyperbaric oxygen therapy by hospital day (HOD) 4 (LVEF 64%; no residual RWMA), consistent with reversible CO-induced myocardial injury.

In the second case (Patient 9), a young adult male patient was brought to our ED after prolonged out-of-hospital cardiopulmonary resuscitation, including multiple defibrillations for ventricular tachycardia, following intentional charcoal-briquette inhalation. On arrival, profound metabolic acidosis (pH 7.09, lactate 14.5 mmol/L) coexisted with massively elevated cardiac biomarkers (troponin I 30.4 ng/mL; CK-MB 169.0 µg/L; CK 5831 U/L). The post-resuscitation electrocardiogram revealed sinus tachycardia with ST-segment depression across the inferior and anterolateral leads, and echocardiography demonstrated severely depressed left ventricular systolic function (LVEF 25%) with regional wall-motion abnormalities not conforming to a single coronary territory. The RWMA pattern was compatible with stress cardiomyopathy. Despite this overt and severe cardiac involvement, the AI-ECG probability score was only 1.0, again misclassifying the patient as low-risk under the manufacturer’s pre-specified threshold. However, the Combined AI model produced a probability of 0.41, well above its operating cutoff, correctly identifying the patient as high-risk. The patient subsequently progressed to brain death.

The Combined AI model correctly reclassified the second, critical case as probability 0.41, whereas it remained discordant in the first case. These observations indicate that the AI-ECG algorithm may underdetect injury patterns characterized by focal RWMA with preserved global function or non-coronary stress cardiomyopathy. Furthermore, they provide direct mechanistic support for the superior discrimination of the Combined AI model (AUC 0.92) over either modality alone. They reinforce that the AI-ECG should be deployed as a complementary digital adjunct among suspected cardiac injury COP patients.

Elevated troponin I levels in COP may result from multiple pathophysiological mechanisms, including hypoxic stress and demand ischemia. In our study, the estimation of CO-CMP using the cardiac biomarker showed a positive predictive value of 47.8% and negative predictive value of 92.9%. These predictive values are conditional on the suspected-cardiac-injury group and should not be extrapolated to unselected ED screening. Elevated troponin I reflects a broad spectrum of myocardial injury, including demand ischemia and transient myocardial stress, which are common in COP and may not necessarily indicate a definitive diagnosis of CO-CMP. The higher specificity of the AI-based model suggests that the ECG-derived digital adjunct predicted myocardial dysfunction more directly related to impaired ventricular contractility. Therefore, although troponin I remains a sensitive screening biomarker, the AI-based model may support early risk stratification by enhancing clinical judgment.

These findings are consistent with prior results demonstrating the diagnostic and prognostic utility of AI-ECG analysis. Previous work has shown that AI-ECG can predict demographic features, reduced LVEF, and silent atrial fibrillation, and that multiple high-risk AI-ECG findings independently predict major adverse cardiovascular events [15]. In comparison, the diagnostic accuracy of the AI-ECG-predicted model for CO-CMP in our study (AUC 0.92) was robust, supporting its potential value as a scalable screening tool in settings where COP remains a prevalent and life-threatening condition.

Notably, earlier approaches to detecting CO-related myocardial dysfunction have relied on composite clinical scoring systems incorporating multiple laboratory and clinical variables. Lee et al. reported excellent discrimination for LVSD using a multivariable clinical score derived from five parameters spanning vital signs, imaging, and laboratory data [24]. Compared with previous multivariable approaches, our model achieved comparable discrimination using a single, standard 12-lead ECG, without requiring additional time-consuming tests. Prior echocardiographic studies have also demonstrated subtle myocardial dysfunction in COP using advanced imaging techniques, such as global longitudinal strain [8]. Although these modalities provide valuable mechanistic insight, they may not be readily available during primary emergency care. By contrast, our findings indicate that AI-ECG analysis can identify clinically meaningful differences in myocardial dysfunction at presentation, reinforcing its role as a rapid and accessible screening tool. However, these findings should be interpreted with caution: 95% bootstrap percentile CI (0.71–1.00) of the optimism-corrected AUC (0.87) indicates susceptibility to overfitting in the current small dataset, and the model is not yet ready for routine clinical use without further external validation.

Our study had several limitations. First, the relatively small sample size and single-center design limit the generalizability of our findings. Our study cohort represents only 4.3% (51/1192) of the CO-poisoned patients during the study period, with the remaining 95.7% not meeting criteria for suspected cardiac injury, and therefore were not referred for cardiology consultation or echocardiography. The AI-ECG model and the multivariable Combined model presented here are therefore applicable to patients with suspected cardiac injury. However, given the rarity and high mortality of CO-CMP, studies focusing on early detection may still offer important clinical insights, despite limited cohort sizes. The small number of outcome events limited the robustness of multivariable modeling. Accordingly, the present Combined model should not be interpreted as a clinically deployable diagnostic tool, but rather as a preliminary exploratory framework for future validation studies. Second, the lack of serial ECG and echocardiographic data precluded assessment of temporal changes in AI-ECG probability scores with myocardial recovery. Third, long-term outcomes could not be evaluated because of limited follow-up data. Nevertheless, even within these constraints, AI-derived ECG-based estimation of left ventricular dysfunction emerged as an independent predictor of CO-CMP, supporting its potential role as an early risk stratification tool in patients with acute COP. Fourth, the AI-ECG model was originally developed and externally validated to detect left ventricular systolic dysfunction (LVSD). However, the definition of CO-CMP in the present study was heterogeneous and included not only reduced LVEF, but also new regional wall-motion abnormalities and right ventricular dysfunction. Therefore, the AI-ECG model should primarily be interpreted as a tool for estimating LVSD-related risk rather than as a comprehensive detector of all phenotypes of CO-induced cardiomyopathy. In particular, its ability to identify isolated regional wall-motion abnormalities or right ventricular dysfunction remains uncertain, because these phenotypes were not specific target conditions during the original model development and validation process. Accordingly, the observed diagnostic performance in the present study may predominantly reflect the model’s ability to detect LV systolic impairment, and the findings should be interpreted as exploratory rather than definitive evidence of broad CO-CMP detection capability.

Importantly, additional sensitivity analysis restricted to patients with isolated LV systolic dysfunction demonstrated generally consistent findings, supporting that the observed association was not exclusively driven by the broader composite definition of CO-CMP. Nevertheless, because of the limited sample size, these findings should still be interpreted cautiously. Accordingly, the present model should not be interpreted as a clinically deployable diagnostic tool, but rather as a preliminary exploratory framework requiring external multicenter validation.

## 5. Conclusions

In this exploratory single-center cohort of CO-poisoned patients with suspected cardiac injury, AI-based ECG analysis demonstrated a potential association with LV systolic dysfunction observed in CO-CMP. Although external validation in larger multicenter cohorts is required, these findings suggest that AI-ECG analysis may provide incremental information for early cardiac risk stratification in selected patients.

## Figures and Tables

**Table 1:** Baseline characteristics.

	CMP (n = 13)	Non-CMP (n = 38)	*p*-Value
Age, years	39.5 ± 17.6	46.6 ± 16.8	0.22
Male sex, n (%)	8 (61.5)	21 (55.3)	0.77
Intentional exposure	12 (92.3)	18 (47.4)	0.005
Hypertension	3 (23.1)	6 (15.8)	0.68
Ejection fraction	40.0 ± 13.8	63.8 ± 6.2	<0.001
Probability score (AI)	11.3 [3.8–32.7]	0.5 [0.2–2.2]	<0.001
COHb (%)	7.5 [4.3–11.4]	4.7 [2.0–20.9]	0.56
Troponin I (ng/mL)	2.37 [0.32–7.88]	0.06 [0.06–0.95]	0.002
CK-MB (ng/mL)	26.6 [16.6–149.0]	2.2 [0.5–21.0]	<0.001
BUN (mg/dL)	21.0 [16.8–35.0]	15.0 [12.6–21.0]	0.011
Creatinine (mg/dL)	1.7 [1.2–2.4]	0.9 [0.7–1.1]	<0.001
Ventilator care	10 (76.9)	8 (21.1)	<0.001
ICU admission	12 (92.3)	16 (42.1)	0.003

Data are presented as mean ± standard deviation, median [interquartile range], or n (%). CMP, cardiomyopathy; COHb, carboxyhemoglobin; CK-MB, creatine kinase myocardial band; CK, creatine kinase; BUN, blood urea nitrogen; ICU, intensive care unit.

**Table 2:** Diagnostic performance for predicting carbon monoxide-induced cardiomyopathy.

	AUC (CI)	Cut-Off	Sensitivity/Specificity	Youden Index	PPV/NPV
AI probability score (manufacturer cutoff)	0.85 (0.70–0.96)	9.7 *	0.54/0.95	0.49	77.8/85.7
AI probability score	0.85 (0.70–0.96)	3.8	0.77/0.82	0.59	58.8/91.2
Cardiac marker model	0.82 (0.70–0.94)	0.19	0.85/0.74	0.58	52.4/93.3
Combined AI model	0.92 (0.83–0.99)	0.15	0.92/0.82	0.74	63.2/96.9

Cardiac marker model: TnI + CK-MB; Combined AI model: sensitivity and specificity were calculated from receiver operating characteristic analysis at the optimal cut-off value. Troponin I and CK-MB were analyzed as continuous predictors in logistic regression and ROC analyses to account for the directionality and magnitude of biomarker-associated risk. * AiTiALVSD pre-specified threshold of 9.7; externally validated for its labeled LVSD. AUC, area under the curve; PPV, positive predictive value; NPV, negative predictive value.

## Data Availability

The datasets used and/or analyzed during the current study are available from the corresponding author on reasonable request.

## Supplementary Materials

The following supporting information can be downloaded at: https://www.mdpi.com/article/10.3390/medicina62061081/s1, Table S1: Echocardiography results of carbon monoxide-induced cardiomyopathy; Table S2: Robustness analyses; Table S3: Sensitivity analysis (isolated LVSD).

## Abbreviations

The following abbreviations are used in this manuscript:

CO	Carbon monoxide
COP	Carbon monoxide poisoning
CO–Hb	Carboxyhemoglobin
ED	Emergency department
CO-CMP	CO-induced cardiomyopathy
CK-MB	Creatine kinase myocardial band
AI	Artificial intelligence
ECG	Electrocardiogram
LVSD	Left ventricular systolic dysfunction
LVEF	Left ventricular ejection fraction
ICU	Intensive care unit
BUN	Blood urea nitrogen
Cr	Serum creatinine
OR	Odds ratio
CI	Confidence interval
ROC	Receiver operating characteristic
AUC	Area under the curve
