# Supplementary material for: Artificial Intelligence-Derived Electrocardiogram Analysis for Identification of Carbon Monoxide-Induced Cardiomyopathy: A Retrospective Study
Source: Medicina (Kaunas). 2026 Jun 2;62(6):1081. doi: 10.3390/medicina62061081 (PMC13304023; doi:10.3390/medicina62061081)
Supplement: Supplementary file 1 [file medicina-62-01081-s001.zip › medicina-4305737-supplementary.pdf]

Table S1. Echocardiography results of carbon monoxide induced cardiomyopathy.

CMP patients	1	2	3	4	5	6	7	8	9	10	11	12	13
Probability score(%)	0.4	0.5	4.7	3.8	7.1	79.5	32.7	14.6	1	29.4	45.2	11.3	41.3
LVEDD (mm)	45	47	51	54	51	50	45	53	40	29	53	47	42
LVESD (mm)	32	30	37	40	41	46	35	48	35	21	37	39	35
IVSd (mm)	7	8	10	9	10	7	9	8	8	8	10	8	11
IVSs (mm)	11	13	13	10	11	9	11	10	11	13	16	11	12
PWd (mm)	7	8	11	8	9	8	9	9	10	8	10	8	10
PWs (mm)	10	14	17	14	12	9	11	10	14	13	15	11	13
RWT	0.31	0.34	0.41	0.31	0.37	0.3	0.4	0.32	0.45	0.55	0.38	0.34	0.5
LVEF (%)	55	48	53	45	40	21	40	16	25	57	55	35	30
LVFS (%)	29	36	27	26	20	8	22	9	12	28	30	17	17
RWMA													
Base_AS	1	1	2	1	2	1	2	2	3	1	1	2	2
Base_Ant	1	1	1	1	2	1	2	2	3	1	1	2	2
Base_AL	1	1	1	1	2	1	2	2	2	1	1	2	2
Base_PL	1	1	1	1	2	1	2	2	2	1	1	2	2
Base_Inf	1	1	1	1	2	1	2	2	2	1	1	2	2
Base_IS	1	1	1	1	2	1	2	3	3	1	1	2	2
Mid_AS	1	1	2	1	2	3	2	3	3	1	1	2	2
Mid_Ant	1	1	2	1	2	3	2	3	3	1	1	2	2
Mid_AL	1	1	1	1	2	3	2	2	2	1	1	2	2
Mid_PL	1	1	1	1	2	3	2	2	2	1	1	2	2
Mid_Inf	1	1	1	1	2	3	2	2	2	1	1	2	2
Mid_IS	1	1	1	1	2	3	2	2	2	1	1	2	2
Apex_Sept	3	3	1	1	2	3	2	3	1	1	2	2	2
Apex_Ant	1	1	1	1	2	3	2	3	1	1	1	2	2
Apex_Lat	1	3	1	1	2	3	2	3	1	1	1	2	2
Apex_Inf	1	3	1	1	2	3	2	3	1	1	2	2	2
RV contractility	1	1	1	1	1	1	1	1	1	2	1	1	1

Left Ventricular End-Diastolic Diameter; LVESD, Left Ventricular End-Systolic Diameter; IVSd, Interventricular Septum thickness at end-Diastole; IVSs, Interventricular Septum thickness at end-Systole; PWd, Posterior Wall thickness at end-Diastole; PWs, Posterior Wall thickness at end-Systole; RWT, Relative Wall Thickness; LVEF, Left Ventricular Ejection Fraction; LVFS, Left Ventricular Fractional Shortening; RWMA, Regional Wall Motion Abnormality; 1,normokinesia; 2, hypokinesia; 3, akinesia; 4, dyskinesia; 5, aneurysm.

Supplementary Table S2. Robustness analyses for the multivariable Combined AI model: standard maximum-likelihood logistic regression, Firth-penalized logistic regression, and bootstrap internal validation (1,000 resamples).

A. Logistic regression coefficients — standard maximum-likelihood vs Firth-penalized.

Variable	β (SE)	OR	95% CI	p-value	Estimation method
Intercept	−2.430 (0.567)	0.088	0.029 – 0.268	<0.001*	Standard ML
AI probability score	0.129 (0.054)	1.138	1.023 – 1.265	0.017*	Standard ML
Troponin I	0.040 (0.052)	1.041	0.940 – 1.153	0.445	Standard ML
CK-MB	0.004 (0.004)	1.004	0.997 – 1.012	0.249	Standard ML
Intercept	−2.200 (0.509)	0.111	0.035 – 0.277	<0.001*	Firth-penalized
AI probability score	0.101 (0.039)	1.107	1.034 – 1.246	<0.001*	Firth-penalized
Troponin I	0.026 (0.039)	1.027	0.953 – 1.138	0.512	Firth-penalized
CK-MB	0.004 (0.003)	1.004	0.997 – 1.011	0.216	Firth-penalized

B. Bootstrap internal validation of model discrimination.

**Metric**	**Value**
Apparent AUC (full sample, standard logistic)	0.921
Apparent AUC (full sample, Firth-penalized)	0.923
Mean bootstrap AUC (in-sample, training)	0.907
Mean bootstrap AUC (applied to original sample, test)	0.855
Mean optimism (training − test)	0.052
Optimism-corrected AUC	0.869
95 % bootstrap percentile CI for corrected AUC	0.709 – 1.006
Bootstrap 95 % CI for AI coefficient (β)	0.063 – 1.003
Successful resamples	1,000 / 1,000

The Combined AI model (AI probability score + troponin I + CK-MB) was fit on (n = 51) patients with 13 CO-CMP events (events-per-variable [EPV] = 4.33). The Firth-penalized odds ratio for the AI probability score was 1.107 (95 % CI 1.034 – 1.246; PLR < 0.001), consistent in direction and magnitude with the standard maximum-likelihood estimate (OR 1.138, 95 % CI 1.023 – 1.265; p = 0.017. Internal validation by ordinary non-parametric bootstrap (B = 1,000 resamples with replacement) was used to estimate optimism in apparent model discrimination. The AUC of 0.921 was reduced to an optimism-corrected AUC of 0.869 after subtracting the mean optimism of 0.052, confirming that the model retains good discriminative performance after accounting for over-fitting. AUC, area under the receiver operating characteristic curve; β, regression coefficient; CI, confidence interval; CK-MB, creatine kinase–MB; CO-CMP, carbon monoxide–induced cardiomyopathy; EPV, events-per-variable; ML, maximum-likelihood; OR, odds ratio; PLR, profile likelihood-ratio p-value; SE, standard error.

Table S3. Sensitivity analysis restricted to isolated left ventricular systolic dysfunction (LVEF <50%).

Variable / Model	Estimate	95% CI	p-value
**AI-only model**			
**- AUC**	0.83	0.68–0.96	—
- **AI probability score (per 1-point increase)**	OR 1.06	1.02–1.13	<0.001
**Combined model**			
**- AUC**	0.92	0.84–0.99	—
- **AI probability score**	OR 1.05	1.01–1.12	0.01
- **Troponin I**	OR 1.02	1.00–1.05	0.08
- **CK-MB**	OR 1.01	1.00–1.01	0.11
**Model comparison**			
**Combined model vs. AI-only model**	ΔAUC 0.09	—	0.15
**Combined model vs. biomarker-only model**	ΔAUC 0.10	—	0.07

Abbreviations: AUC, area under the receiver operating characteristic curve; CI, confidence interval; CK-MB, creatine kinase-myocardial band; LV, left ventricular; LVEF, left ventricular ejection fraction; OR, odds ratio. Sensitivity analysis restricted to patients with isolated LV systolic dysfunction (LVEF <50%) demonstrated generally consistent findings with the primary composite CO-CMP analysis, supporting that the observed association was not solely driven by the broader composite endpoint definition.
